# Flow patterns through side holes of venous cannula in mock circulation loop

**DOI:** 10.1097/MD.0000000000045016

**Published:** 2025-10-24

**Authors:** Dong Hoon Kang, Jong Woo Kim, Seong Ho Moon, Sang Kwon Lee, Ho Jeong Cha, Joung Hun Byun

**Affiliations:** a Department of Thoracic and Cardiovascular Surgery, Gyeongsang National University College of Medicine, Gyeongsang National University Changwon Hospital, Changwon, Republic of Korea.

**Keywords:** cannula, cannula, extracorporeal membrane oxygenation, pump flow rate

## Abstract

Cannulae with multi-staged side holes (SHs) for venous drainage are widely used for performing extracorporeal circulation. Various types of catheters are used worldwide; however, data on the flow performance of each catheter are lacking or inaccurate. Clinical applications are often intuitive. In this study, we aimed to develop a mock circulation loop for cannula performance evaluation. We found no differences in the rate of change in the flow rate through each SH as the pump flow increased when the pump flow rate was 250 to 750 mL/min. When the pump flow rate was ≥ 750 mL/min, there was an increase in the rate of change in the flow rate through each side hole toward the proximal side hole and the percentage inflow of the proximal side hole and decrease in percentage inflow toward the distal side hole, as the pump flow increased. Thus, we believe that the desired venous drainage can be applied to patients, and the fluid dynamical structures of catheters in the human body can be estimated according to the type of catheter or its location in the human body. The findings of this study will aid in designing new catheters.

## 1. Introduction

Peripheral cannulation via the femoral vessels for extracorporeal membrane oxygenation (ECMO) or cardiopulmonary bypass (CPB) in patients with heart or lung failure has been established.^[1,2]^ Recognizing the various aspects of flow through the catheter inserted into the vena cava is essential for successful treatment of the patient, depending on the purpose for which extracorporeal circulation is being used. In other words, it is important to know how much blood can be drained at any point in the vena cava or right atrium using the current catheter and the extent to which the flow rate of the main pump will be more effective. This is an important concern in improving the clinical situation of patients. The flow performance of a venous cannula is mainly affected by its diameter and length, as well as several other geometrical factors, such as side holes (SHs).^[3,4]^ A recent study suggests that subtle changes in cannula geometry (specifically, the size, spacing, and angle of SHs) can significantly improve internal blood flow dynamics, thereby substantially reducing the risk of thrombosis.^[5]^ Despite the importance of information on the cannula’s performance, only limited knowledge is available regarding the impact of SH parameters, such as amount, arrangement, location, shape, or size on the flow performance. Recently, due to advances in computational fluid dynamics, studies on complex flow patterns have become more accessible, but the reliability of flow simulation is still not enough to implement. Oversimplification of computational models often leads to biased or misleading results.^[6]^ In this study, we measured and graphed the actual flow rate of the fluid through the SHs of the catheter. To the best of our knowledge, only a few studies have measured the flow patterns through the SHs of catheters. Although the mock circulation loop setup applied to this study could not satisfy all the complex conditions in the actual human body, the oversimplification that could cause insufficient results was minimized. In this study, we aimed to determine the drainage performance of the SH of a cannula at different flow rates and help identify more accurate flow patterns of the catheters currently in use.

## 2. Materials and methods

Ethical approval was waived for this study because no patients or animals were included, and only an artificial circuit was used.

### 2.1. Mock circulation loop setup

**Figure 1.**
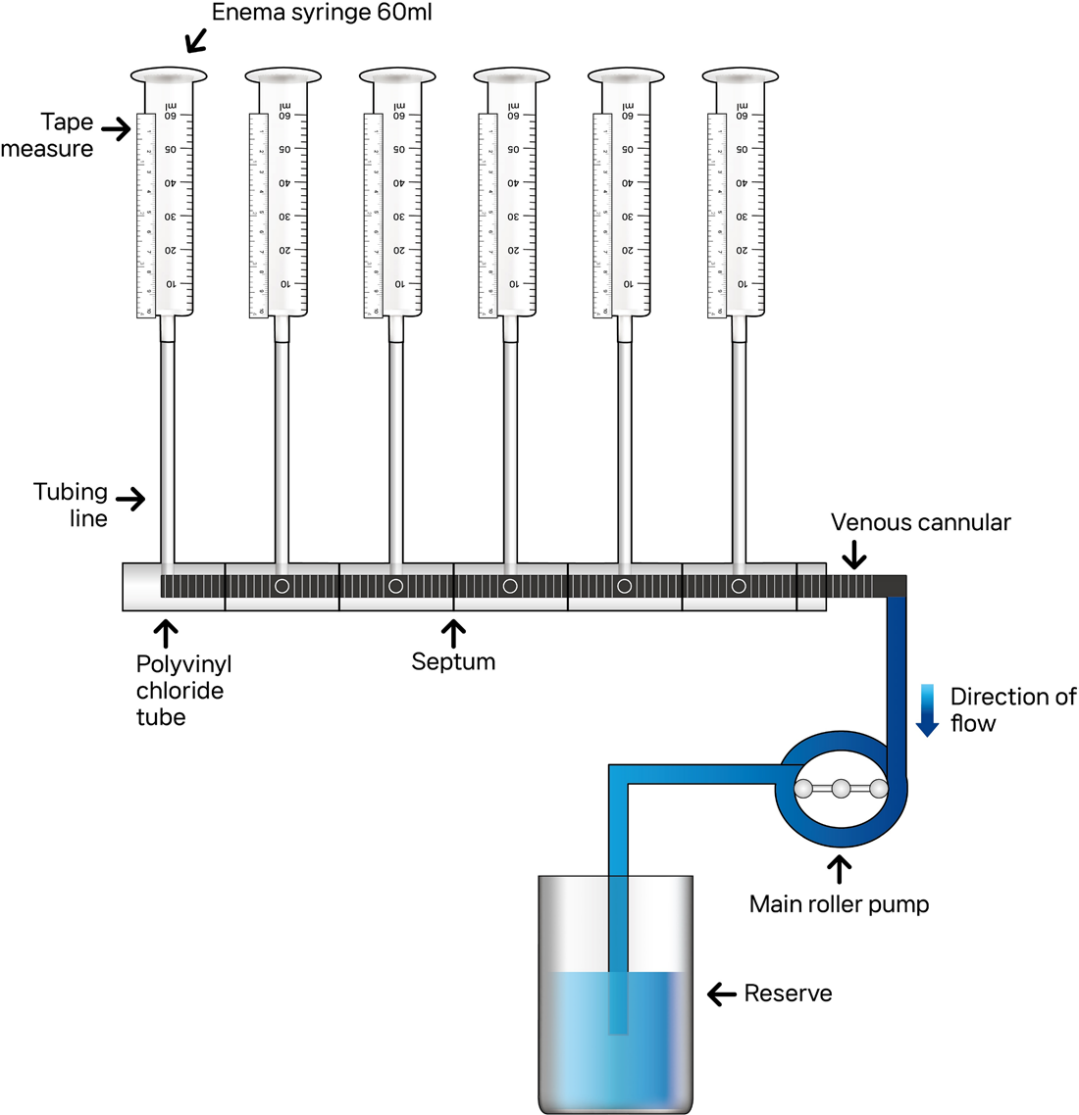
The setup of the mock loop utilized in the study. The mock loop consisted of a femoral venous cannula with 20 side holes, which was inserted into a rigid polyvinyl chloride tube to simulate the vena cava.

Figure 1 represents the setup of the utilized mock loop. It consisted of a femoral venous cannula (23 Fr 55 cm; MAQUET HLS Cannulae; Table 1) with 20 SHs that was inserted into a rigid polyvinyl chloride tube (length, diameter) assumed to be the vena cava. Each SH was arranged at 90° intervals; thus, 4 SHs formed 1 pair, and 5 pairs were arranged in the catheter. The interior of the rigid tube was equipped with a chamber to enable each SH to function independently. The most distal SH was designated as 1, and the rest were numbered 2, 3, 4, and 5, going toward the proximal side. Using a tubing line (3/8 × 3/32 RAUMADIC ECC no DOP blood Line Tubing), 6 enema syringes of 60 mL each were connected to each part of the rigid tube where each SH was located. The circuit was driven by a roller pump (6-inch pump; Code century Heart Lung machine) with constant flow.

**Table 1:** Dimensions of catheter.

Cannula length	550 mm
Cannula inner diameter	7.37 mm
Cannula thickness	0.33 mm
Side hole diameter	2.5 mm
Side hole interval	37 mm
Side hole number	20
Distance from end tip opening to 1st side hole	41 mm

### 2.2. Measurement of the flow rate through catheter SHs

According to our preliminary measurement, the 1.5-cm height of a 60-mL enema syringe has a volume of approximately 10 mL. After installing a tape measure outside each syringe and starting the main pump, a video was taken to measure the moving distance of the water surface. After taking 10 shots at each pump flow rate, the change in the height of the water surface in the syringe for 1 second was measured in the still frame of the filmed video, and the average was obtained. The flow rate through SHs of catheter could be calculated by the following equation:


$$\begin{array}{l} \begin{array}{llll} & The\;flow\;rate\;through\;catheter\;SHs\;according\;\; & to\;the\;roller\;pump\;flow\;rate\;\left(mL/min\right)\; & =\frac{10\left(mL\right)\times Water\;moving\;distance\;during\;1\;s\;\left(cm/s\right)\times 60\;\left(s\right)}{1.5\;cm}\; \end{array} \end{array}$$

### 2.3. Statistical analysis

Continuous variables are expressed as means ± standard deviations. The comparisons between groups (the rate of change in the flow rate through each SH) were performed with the Mann–Whitney test and the Kruskal–Wallis test of differences. Multivariate logistic regression analyses were not performed to determine the rate of change in the flow rate through each SH. All statistical analyses were performed using SPSS software version 24.0 (IBM Corp., Armonk). A *P* value < .05 was considered statistically significant.

## 3. Results

**Figure 2.**
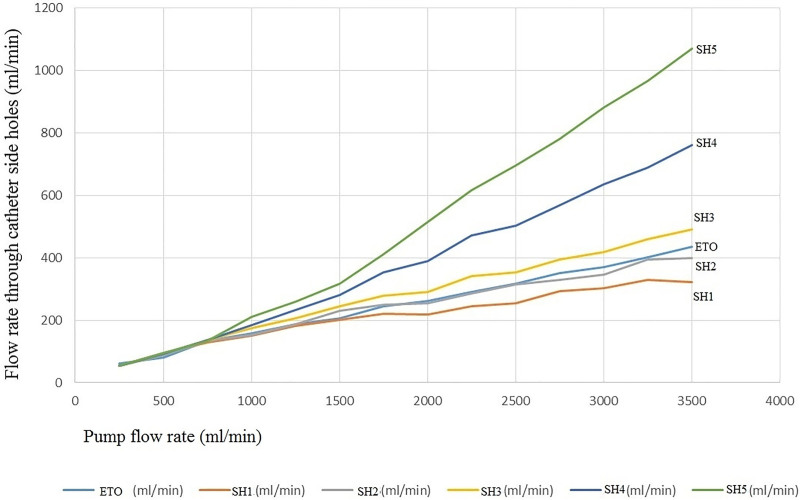
The flow rate through each side hole according to the pump flow.

**Figure 3.**
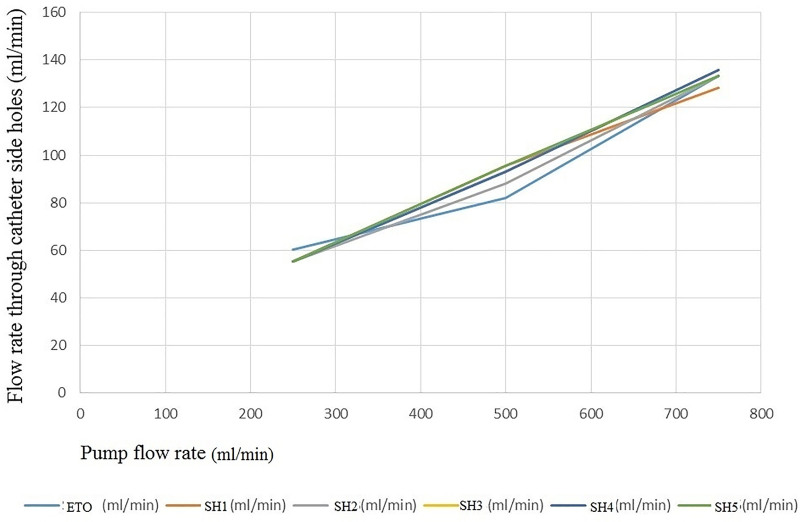
When the pump flow rate was between 250 and 750 mL/min, as the pump flow increased, the rate of change in the flow rate (the slope of a line) through each side hole was almost same.

The flow rate through catheter SHs according to the pump flow rate are shown in Table 2 and Figures 2 and 3. When the pump flow rate was between 250 and 750 mL/min, as the pump flow increased, the rate of change in the flow rate through each SH (change in flow rate through the SH/pump flow change) exhibited no differences according to the Mann–Whitney test (*P* = .065; Fig. 3). Moreover, when the pump flow rate was between 250 and 750 mL/min, the flow rate through each SH was almost the same. When the pump flow rate was ≥ 750 mL/min, the rate of change in the flow rate through each SH increased toward the proximal SH as the pump flow increased.

**Table 2:** Measured flow rate through catheter side holes according to the roller pump flow rate.

Pump flow rate (mL/min)	ETO (mL/min)	SH 1 (mL/min)	SH 2 (mL/min)	SH 3 (mL/min)	SH 4 (mL/min)	SH 5 (mL/min)
250	60.3 ± 7.16	55.28 ± 4.74	55.28 ± 4.74	55.28 ± 4.74	55.28 ± 4.74	55.28 ± 4.74
500	81.91 ± 7.1	95.48 ± 9.3	87.94 ± 10.4	92.96 ± 10.4	92.96 ± 10.4	95.45 ± 9.3
750	133.16 ± 10.4	128.14 ± 10.4	133.16 ± 10.4	135.68 ± 9.3	135.68 ± 9.3	133.16 ± 10.4
1000	158.79 ± 14.83	150.75 ± 10.59	152.76 ± 10.38	174.87 ± 9.7	184.92 ± 8.47	211.05 ± 17.08
1250	186.93 ± 9.7	182.91 ± 6.36	186.93 ± 13.56	207.03 ± 9.7	233.16 ± 10.38	259.29 ± 19.9
1500	205.2 ± 12.7	201 ± 16.4	231.18 ± 12.71	245.22 ± 22.8	281.4 ± 37	317.58 ± 35.2
1750	245.22 ± 12.7	221.1 ± 21.18	249.24 ± 16.95	277.38 ± 22.82	353.76 ± 16.95	410.04 ± 25.42
2000	261.3 ± 21.19	217.08 ± 20.76	253.76 ± 33.1	289.44 ± 16.95	389.94 ± 27.13	514.3 ± 25.33
2250	289.44 ± 16.95	245.22 ± 22.82	285.42 ± 22.82	341.7 ± 21.19	470.3 ± 27.03	615.06 ± 27.13
2500	317.58 ± 12.71	253.26 ± 19.42	313.56 ± 16.9	353.76 ± 16.9	502.28 ± 20.9	695.46 ± 19.4
2750	349.74 ± 27.1	293.46 ± 19.4	329.64 ± 16.9	393.96 ± 31.7	566.7 ± 29.8	779.88 ± 38.8
3000	369.84 ± 31.7	301.5 ± 21.2	345.72 ± 20.8	418.08 ± 28.1	635.16 ± 36.9	880.38 ± 35.2
3250	402 ± 0	329.64 ± 16.9	393.96 ± 16.9	458.28 ± 20.7	687.42 ± 12.7	964.8 ± 26.8
3500	434.16 ± 25.4	321.6 ± 18.9	397.98 ± 12.7	490.44 ± 25.4	759.78 ± 35.1	1069.32 ± 33.8

Data expressed as mean ± standard deviation.

ETO = end tip opening, SH = side hole.

**Figure 4.**
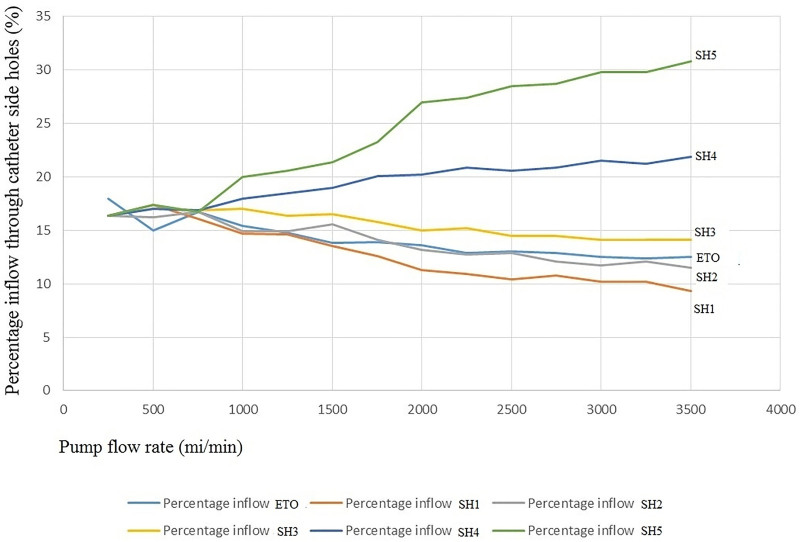
The percentage of inflow through each catheter side hole according to the pump flow rate.

Percentage inflow through each catheter SH and according to the pump flow rate are shown in Table 3 and Figure 4. When the pump flow rate was between 250 and 750 mL/min, as the pump flow increased, the percentage inflow through each SH did not differ according to the Kruskal–Wallis test (*P* = .614). When the pump flow rate was ≥ 750 mL/min, the percentage inflow of the proximal SH 4, 5 increased as the pump flow rate increased, and the percentage inflow decreased toward the distal SH 1, 2, 3, and end tip opening.

**Table 3:** Percentage inflow through each catheter side hole according to the roller pump flow rate.

Pump flow rate (mL/min)	ETO (%)	SH 1 (%)	SH 2 (%)	SH 3 (%)	SH 4 (%)	SH 5 (%)
250	18	16.4	16.4	16.4	16.4	16.4
500	15	17.4	16.2	17	17	17.4
750	16.7	16.1	16.7	16.9	16.9	16.7
1000	15.4	14.7	14.9	17	18	20
1250	14.8	14.6	14.9	16.4	18.5	20.6
1500	13.8	13.5	15.6	16.5	19	21.4
1750	13.9	12.6	14.1	15.8	20.1	23.3
2000	13.6	11.3	13.2	15	20.2	27
2250	12.9	10.9	12.7	15.2	20.9	27.4
2500	13	10.4	12.9	14.5	20.6	28.5
2750	12.9	10.8	12.1	14.5	20.9	28.7
3000	12.5	10.2	11.7	14.1	21.5	29.8
3250	12.4	10.2	12.1	14.1	21.2	29.8
3500	12.5	9.3	11.5	14.1	21.9	30.8

ETO = end tip opening, SH = side hole.

## 4. Discussion

Fluid dynamical structures of catheters in the human body are very complex. In clinical situations where ECMO or CPB are applied, many complex factors, such as blood viscosity, vessel diameter, blood pressure, cannula diameter, and vessel-to-cannula diameter ratio, must be considered. Since implementing all these factors was very difficult, we simplified the complexity and conducted this study. Although our model differs from the fluid dynamical structures of a catheter in the human body, we believe that our model can provide useful information to understand the flow patterns through the SHs of the catheter.

When the main pump flow rate was less than approximately 750 mL/min, as the pump rate increased, the flow rate through all SHs increased uniformly regardless of the position of the SH. At the same main pump flow rate, the flow rate through each SH was almost the same. If the increased interval of the main pump flow rate is small, the flow rate through the SH may be constant in any section > 750 mL/min. Although further research is required, it is worthwhile to know that the flow rate patterns through the SHs at the lower flow rate of the main pump are constant (Figs. 2 and 3, Table 2). To the best of our knowledge, only a few studies have shown these flow patterns of SHs. If this phenomenon occurs in the human body, it is assumed that the fluid flow in the catheter will be in straight streamlines at a lower flow rate than that of the main pump (≤800 mL/min), and the risk of thrombosis in all the SHs will be the same.

In particular, Figure 4 shows that as the pump flow increases at the lower flow rate, there is no difference in the percentage inflow through each SH. Therefore, even if the number of SHs is increased at the same pump flow rate, the total flow is not affected. Rather, the mean shear rate through each SH decreases,^[6]^ and the flow rate through the SH also decreases; thus, the risk of thrombosis may increase in all the SHs.^[7–10]^ At a higher pump rate of the main pump (≥800 mL/min), as the pump rate increased, a greater amount of fluid was moved through the SH located on the proximal side. This can give us important information regarding the placement of the catheter to obtain the clinically necessary blood drainage pattern, even though blood was not used in this study.

In clinical practice, we sometimes place the distal end of the catheter close to the superior vena cava (SVC) or SVC–right atrium (RA) junction through the femoral vein to effectively drain blood from the SVC when a drain catheter is inserted. However, in reality, the SVC blood may not be effectively drained through the distal SH of the catheter; instead, SVC blood enters the RA and mixes with the blood from the inferior vena cava. Thereafter, the venous blood either drains through the SHs of the catheter in the RA or through the proximal SHs near the inferior vena cava–RA junction, depending on the main pump flow rate.

It is thought to be helpful in indirectly estimating the patterns of recirculation during venovenous ECMO and determining the appropriate location of the catheter for effective venous drainage during CPB. Moreover, if we insert a venous drainage catheter through the right internal jugular vein, we can infer the movement patterns of blood through the SHs based on the position of the catheter. As shown in Figure 3 and Table 3, as the pump rate increases in the higher pump rate of the main pump (≥800 mL/min), the percentage inflow increases in proximal SH 4, 5 and gradually decreases in distal SHs. If the pump flow is increased, a large fraction of the total fluid moving into the catheter will pass through the proximal SHs.

As the main pump flow increases, the overall shear rate increases, but the rate of change of the shear rate in the proximal SHs is greater than that of the distal SHs. As a result, the formation of thrombi may occur relatively more easily in the distal part of the catheter than in the proximal part. Low shear rate values may cause the formation of thrombi,^[11]^ platelet adhesion and aggregation,^[12,13]^ whereas high shear stress values are responsible for potential red blood cell and platelet destruction.^[14–16]^

Although we did not replicate the actual scenario of the human body and did not use computational simulation in this study, this study can provide important information for future studies on the fluid mechanics of the catheter and movement patterns of blood through the catheter in the human body.

### 4.1. Limitation

In this study, water was used instead of blood; however, no major issues in recognizing the overall flow patterns were noted. A previous study demonstrated that changes in blood viscosity did not affect velocity at a given flow rate in a fixed-diameter vessel with laminar flow, which can be explained by the Fahraeus–Lindquist effect.^[17]^ In our mock circulatory loop setup, the flow around the catheter was stopped. In the actual human body, the blood around the catheter flows. In practice, fluid flow around these catheters may affect the flow rate through the SHs. However, if the fluid flow around the catheter is constant, there may be no difference in the flow patterns even though the flow rate through the SHs changes slightly. Further studies using blood or employing real patients could validate our study results.

We started the main pump to measure the distance the water surface moved, installed a tape measure outside each syringe, and recorded a video. We believe that errors may have occurred while measuring the distance of water surface movement. To reduce any measurement error, measurements were repeated 10 times for each pump flow rate. The error may be reduced because the moving distance of the water surface was measured in the still frame of the filmed video. This may inevitably be different from the precise fluid dynamical structures of a catheter in the human body because it cannot actualize the situation of blood flow in the human body.

The results obtained in our study were from a single type of catheter. If the same experiment is performed using a different type of catheter, different results may be garnered. Therefore, if research using other types of catheters is conducted in the future, it may help design catheters for proper drainage.

## 5. Conclusion

Applying the flow patterns of SHs demonstrated in our study may benefit patients requiring ECMO or CPB. The necessary venous drainage aspects can be applied to patients and may be very helpful in estimating the fluid dynamical structures of catheter in the human body according to the type of catheter or its location. Moreover, this study can optimize new cannulas.

## Acknowledgments

The authors are grateful to Lim Han Jung, Yoo Moon Sik and Choi Ji Hun, Park Jae Jin, Kim Geon Woo, Cha Jun A, Seo Ji Won from Gyeongsang National University Changwon Hospital for their perfusionist and physical assistant. And we extend our sincere gratitude to Dr Seok Joong Kang, a specialist in fisheries science, for their invaluable contribution in fabricating the mock circulation loop.

## Author contributions

**Conceptualization:** Joung Hun Byun.

**Data curation:** Jong Woo Kim, Seong Ho Moon, Sang Kwon Lee, Ho Jeong Cha.

**Formal analysis:** Dong Hoon Kang, Joung Hun Byun.

**Methodology:** Dong Hoon Kang, Joung Hun Byun.

**Supervision:** Joung Hun Byun.

**Writing – original draft:** Dong Hoon Kang, Joung Hun Byun.

**Writing – review & editing:** Joung Hun Byun.
